# A Standardized Prism-Based TIRF Platform for Quantitative Single-Molecule Fluorescence Studies of Biomolecular Dynamics

**DOI:** 10.3390/bios16060331

**Published:** 2026-06-10

**Authors:** Arijit Patra, Lunden Melton, Lenwood S. Sawyer, Tate King, Sujay Ray

**Affiliations:** Department of Chemistry and Biochemistry, University of Mississippi, Oxford, MS 38677, USA

**Keywords:** prism-based TIRF (pTIR), prism-type total internal reflection fluorescence microscopy, single-molecule fluorescence, smFRET, evanescent wave, microscope construction, optical alignment, flow-cell preparation, surface immobilization, quantitative performance benchmarks, photobleaching statistics, signal-to-noise ratio, donor leakage, single-molecule data analysis

## Abstract

Single-molecule Förster resonance energy transfer (smFRET) enables direct measurement of nanoscale conformational dynamics and heterogeneity in biomolecules, but quantitative interpretation of smFRET data critically depends on well-controlled excitation geometry, low background fluorescence, robust calibration, and reproducible data-analysis workflows. Prism-based total internal reflection fluorescence (pTIRF) microscopy provides important advantages for such measurements by physically separating excitation and emission paths and generating a highly confined evanescent field, yet practical guidance for implementing reproducible, quantitative pTIRF systems remains fragmented. Here we present a comprehensive, standardized framework for the design, alignment, calibration, validation, and operation of a prism-based TIRF microscope optimized for single-molecule fluorescence measurements. We describe the complete optical architecture for dual-color excitation and detection, establish alignment invariants that ensure reproducible evanescent excitation and stable donor–acceptor channel registration, and detail surface preparation, flow control, and photostabilization strategies required for reliable long-term imaging. Quantitative benchmarking protocols are introduced to evaluate signal-to-noise ratio, photobleaching kinetics, and spectral crosstalk, providing objective criteria for defining optimal operating conditions and instrument performance limits. Finally, we integrate these experimental procedures with an end-to-end single-molecule data-analysis workflow encompassing channel registration, automated and manual trajectory selection, FRET calculation, and kinetic analysis using hidden Markov modeling. The utility of the platform is demonstrated through smFRET measurements of conformational dynamics in a model nucleic acid system. Together, this work provides a reproducible and accessible methodology for implementing prism-based TIRF microscopy as a robust quantitative platform for single-molecule fluorescence studies across a wide range of biomolecular systems.

## 1. Introduction

Single-molecule (SM) fluorescence techniques have transformed the study of biomolecular dynamics by enabling direct observation of stochastic molecular behavior that is masked in traditional ensemble measurements [1,2,3,4]. Among these methods, SM-Förster resonance energy transfer (smFRET) provides a powerful approach for monitoring nanometer-scale conformational changes in real time [1,4,5,6,7,8]. Because FRET efficiency depends sensitively on the distance between donor and acceptor fluorophores [3,4,8], smFRET measurements allow the quantitative characterization of structural transitions, conformational heterogeneity, and kinetic intermediates in nucleic acids and proteins [4,5,9,10,11,12,13,14,15,16,17]. Reliable smFRET measurements, however, require excitation geometries [3,18,19,20] that maximize signal-to-background ratio [4,21,22,23] while maintaining stable fluorescence detection over long observation times [3,4,21,22].

Total internal reflection fluorescence (TIRF) microscopy is widely used for this purpose because it generates an evanescent excitation field confined to the vicinity of the glass–water interface, selectively exciting surface-immobilized molecules while suppressing fluorescence from molecules in solution [1,3,4,18,19,23]. Among TIRF implementations, prism-based TIRF (pTIRF) offers several advantages for quantitative SM experiments [4]. In this geometry, excitation light is introduced externally through a prism rather than through the microscope objective, physically separating excitation and emission optical paths [23,24]. This configuration reduces background arising from objective autofluorescence and scattering, while also allowing independent optimization of excitation geometry and emission detection [22,23,24,25]. As a result, pTIRF systems can achieve high signal-to-background ratios and flexible control of excitation parameters, features that are particularly valuable for quantitative smFRET measurements.

Despite these advantages [23,25], pTIRF systems are less commonly implemented than objective-based TIRF configurations [16,26,27]. A major reason is that the practical requirements for building and operating a quantitative pTIRF instrument are rarely described in a unified manner. Published reports [26,27] often present individual aspects of the system—such as optical layouts, sample preparation methods, or data-analysis approaches—but detailed guidance on how these components integrate into a stable and reproducible experimental platform is typically fragmented across the literature. Key aspects such as reproducible excitation geometry, alignment invariants, calibration procedures, and quantitative performance validation are often only briefly described. Therefore, laboratories attempting to establish pTIRF instrumentation frequently rely on trial-and-error optimization, making it difficult to reproduce system performance or compare measurements across experiments and laboratories. For quantitative SM-fluorescence experiments, this lack of standardized methodology presents a significant challenge. Accurate extraction of fluorescence intensities and FRET efficiencies depends not only on the optical configuration of the microscope but also on precise alignment of excitation geometry [18,22,24], stable registration between donor and acceptor detection channels [4,19,20,22], and rigorous calibration of system performance [21]. Without well-defined procedures for establishing and validating these conditions, instrumental artifacts such as background fluctuations, channel misregistration, or excitation variability can propagate directly into SM trajectories and kinetic measurements [4,22,23,28,29,30,31].

To address the lack of standardized guidance for quantitative prism-based TIRF experiments, we present a comprehensive framework for the design, alignment, calibration, and validation of a pTIRF microscope optimized for single-molecule fluorescence measurements. Beyond describing the optical architecture of the system, this work establishes a set of alignment invariants that define reproducible excitation geometry and channel registration, enabling consistent evanescent excitation across experiments. We further introduce quantitative benchmarking procedures that evaluate key performance metrics, including signal-to-noise ratio, photobleaching behavior, and spectral crosstalk between donor and acceptor channels. These measurements define the operational limits of the instrument and provide objective criteria for optimizing imaging conditions. Finally, we integrate these experimental and calibration procedures with a complete single-molecule data-analysis workflow, from raw image acquisition through trace extraction, FRET calculation, and state identification. Application of the platform is demonstrated using single-molecule measurements of conformational dynamics in a model nucleic acid system. Together, this work provides a reproducible methodology for implementing prism-based TIRF microscopy as a quantitative single-molecule measurement platform. While the present study focuses on nucleic acid systems as a benchmark, the platform is broadly applicable to other biomolecular systems including proteins, protein–nucleic acid complexes, and membrane-associated assemblies, provided suitable labeling and immobilization strategies are used.

## 2. Optical Design of the Prism-TIRF Platform

### 2.1. The Excitation Pathway

**Figure 1:**
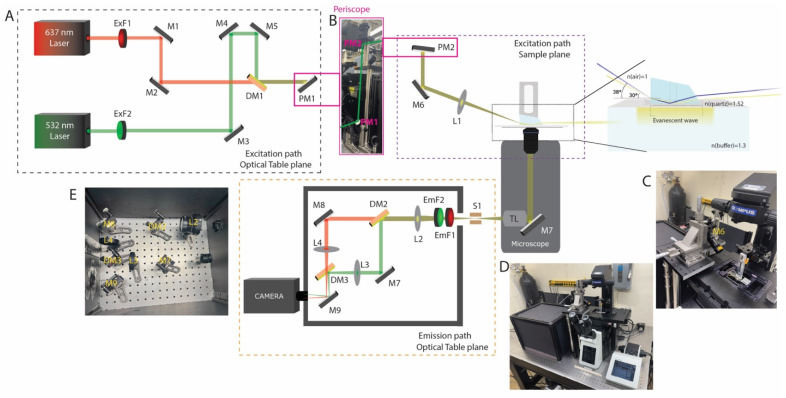
Optical layout and implementation of the dual-color TIRF microscope. (**A**) Schematic of the excitation path on the optical table. Independent 637 nm and 532 nm lasers are filtered (ExF1–ExF2), combined using a dichroic mirror (DM1), and routed through steering mirrors (M1–M5) toward the microscope via a periscope (PM1–PM2). Excitation optics at the microscope. The collimated beam is directed through relay optics (M6, L1) to the prism. The oblique angle creates the TIRF angle. The inset illustrates total internal reflection at the quartz–sample interface, generating an evanescent field in the aqueous buffer. Emission path schematic and photograph. Fluorescence collected by the objective is separated from excitation light, spectrally split by dichroics (DM2–DM3), filtered (EmF1–EmF2), and imaged onto the camera via relay lenses (L2–L4), and mirrors (M7–M9). (**B**) Photograph of the periscope assembly to change the optical plane from the optical table to the sample plane. (**C**) Photograph of the excitation-side microscope assembly, highlighting the objective mount and incidence-angle control optics. (**D**) Photograph of the complete custom-built microscope system integrated with excitation and emission detection paths. (**E**) Photograph of the emission-side microscope assembly in optically isolated box.

Two excitation lasers are employed in the prism-based total internal reflection fluorescence (TIRF) setup: a green laser at 532 nm and a red laser at 637 nm (Figure 1A). The green source is a Sapphire 532 FPT FT (Coherent Corp, Saxonburg, PA, USA) (output power 10 mW–0.5 W; beam diameter 1.5 ± 0.05 mm), and the red source is an OBIS LX 637 (Coherent Corp, Saxonburg, PA, USA) (output power 140–180 mW; beam diameter 0.70 ± 0.1 mm). Although demonstrated with 532 nm and 637 nm excitation, the optical design is modular and can be readily extended to additional wavelengths and fluorophore combinations. The excitation beams are directed toward the microscope using a series of mirrors (Figure 1A). In addition to standard mirrors, coated dichroic beamsplitters are incorporated to combine the red and green optical paths, transmitting the red excitation beam while reflecting the green excitation beam (Figure 1A). The dichroic elements used are T635lpxr-UF2 (Chroma, Bellows Falls, VT, USA), UltraFlat series: UF2 optics (2 mm thickness; ≤0.5 waves/inch peak-to-valley flatness) were selected over UF1 (1 mm thickness; ≤2 waves/inch P–V) due to their superior surface flatness, which is essential for minimizing wavefront distortion in TIRF applications. Total internal reflection is achieved using a mirror to create a near-critical angle of incidence at the interface, as shown in Figure 1B. Finally, a Pellin–Broca prism (refractive index ≈ 1.52) enables generation of an evanescent field at the glass–aqueous interface, selectively exciting fluorophores within a limited distance from the surface (Figure 1B,C). Because the evanescent field decays exponentially with distance, fluorescence is restricted to molecules immobilized near the interface, reducing background from the bulk solution. The penetration depth is governed by the angle of incidence; in this configuration, an incident angle of ~70° yields a calculated penetration depth of approximately 124 nm. Increasing the angle of incidence increases the penetration depth and can elevate background signal; but it also makes beam alignment more difficult and limits the practical ability to achieve such high angles. The angle of incidence and penetration depth were determined using Snell’s law and the standard expression for evanescent field decay (Figure 1B).

The prism is mounted on a custom stage that permits precise positioning in the x, y, and z axes, allowing accurate placement over the sample slide (Figure 1C). Sample alignment is performed using 10×, 20× objectives (numerical apertures 0.30 and 0.50, respectively), data acquisition is performed using a 60× objective (NA = 1.42). The 60× objective has a working distance of approximately 300 µm, allowing efficient collection of fluorescence from the top surface of the sample channel (~0.15 mm for the #1 coverslip + ~0.05 mm for the double-sided tape). Emitted fluorescence is collected through the objective and directed into the detection path via internal microscope optics.

### 2.2. The Emission Pathway

Fluorescence emitted from the sample is collected through the objective and relayed by the microscope tube lens to an intermediate image plane near the camera port (Figure 1B,E). The emission is then passed through a rectangular slit to define the field of view and produce a spatially confined, rectangular beam profile. To enable dual-color detection, the emitted light is collimated and directed onto a dichroic beamsplitter, which reflects wavelengths below 610 nm and transmits wavelengths above 640 nm, thereby separating the signal into two spectrally distinct channels. The two channels are subsequently recombined using a second dichroic element and projected onto spatially separated regions of the camera sensor. Fluorescence detection is performed using an ORCA-Fusion scientific CMOS camera (C14440; Hamamatsu (Hamamatsu City, Japan)), featuring a 6.5 µm × 6.5 µm pixel size and a peak quantum efficiency of ~80%, enabling high-sensitivity detection under low-signal conditions. Residual excitation light at 532 nm and 637 nm is effectively suppressed using a combination of bandpass and long-pass emission filters positioned upstream of the detector, ensuring selective transmission of fluorescence emission while minimizing background contributions. All optical parts used in the setup is listed below in Table 1.

## 3. Alignment Invariants

### 3.1. Stable Evanescent Excitation

In pTIRF, reproducible excitation geometry is important for obtaining stable single-molecule fluorescence data that require several alignment parameters to remain invariant once the system has been optimized. These include the relative beam position at the prism face, the angle of incidence at the quartz–sample interface, the overlap of the excitation region with the objective field of view, and the spatial registration of donor and acceptor emission channels on the camera. Deviation in any of these parameters can lead to inconsistent evanescent excitation, spatial mismatch between channels, or reduction in signal-to-noise ratio. We aligned the excitation beam so that it entered the prism at a defined angle and produced total internal reflection at the quartz slide–aqueous sample interface. To establish a reproducible TIR geometry, the relative positions of the stage mirror, prism, slide chamber, and excitation beam were kept constant. The distance between the prism and the stage mirror was measured to be 22.5 cm, and the height of the mirror from the stage breadboard was set to 13.5 cm. Based on these measurements, the beam path formed a hypotenuse of approximately 26.2 cm. The incident angle at the prism face was estimated from the geometry: tan(θ) = 13.5/22.5, which gives θ~30°. This prism-entry angle leads to a ~20° angle of refraction that further estimated incident angle of approximately 70° at the quartz–aqueous interface, which is suitable for generating total internal reflection. Once this geometry was established, the beam position on the prism and sample chamber was kept unchanged between experiments.

### 3.2. Evanescent Field Characterization

The evanescent field generated in pTIR decays exponentially away from the quartz slide–aqueous interface, allowing for selective excitation of fluorophores located near the slide surface. The evanescent field intensity, I(z), at a perpendicular distance z from the interface, depends proportionally with d, the penetration depth. The penetration depth of this field is determined by the wavelength of the excitation light, the refractive indices of the prism and sample medium, and the angle of incidence at the interface. In the present setup, the incident angle applied to the Pellin–Broca prism was 30° that corresponds to an estimated penetration depth of approximately 124 nm, while an incident angle ~38° leads to a penetration depth of 479 nm. This shallow excitation region minimizes fluorescence from molecules in bulk solutions and thereby lowers background during single-molecule imaging. Characterization of the evanescent field is important for confirming that the microscope is operating under true TIRF conditions rather than in a near-critical or partially epifluorescence regime. One practical indication of proper evanescent excitation is the selective visualization of surface-immobilized fluorophores with minimal diffuse fluorescence from the surrounding solution. When the angle of incidence is decreased away from the TIR condition, increased excitation of fluorophores in solution is observed, producing elevated background in the camera. Thus, optimization of the beam angle at the prism provides both a theoretical and visual method for confirming appropriate field generation. Since penetration depth alters with angle of incidence, this parameter may also be adjusted depending on the application. A shallower field improves surface selectivity, whereas a deeper field may increase total fluorescence signal at the cost of a higher background. Therefore, maintaining a defined angle of incidence during alignment is necessary for obtaining reproducible measurements across experiments. In practice, we kept the selected prism geometry, and beam path preserved once the desired penetration depth and imaging performance had been achieved.

## 4. Slide Preparation and Flow Control

### 4.1. Slide Preparation

**Figure 2:**
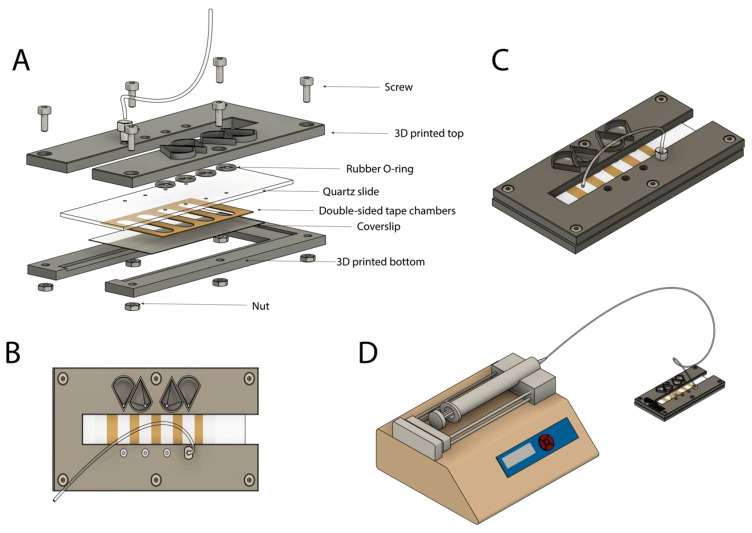
Assembly and operation of the microfluidic flow chamber. (**A**) Exploded view of the custom flow chamber assembly. The device consists of a 3D-printed top and bottom housing clamping a quartz slide and coverslip, separated by double-sided tape defining the microfluidic channels. Rubber O-rings provide fluidic sealing, and the assembly is secured using screws and nuts. (**B**) Top-down view of the assembled chamber showing parallel microfluidic channels formed by patterned adhesive tape and inlet/outlet tubing connections for solution exchange. (**C**) Three-dimensional rendering of the fully assembled flow chamber highlighting its compact footprint and integrated tubing for interfacing with external fluid delivery systems. (**D**) Experimental configuration of the flow chamber connected to a syringe pump for controlled solution flow during imaging experiments.

**Figure 3:**
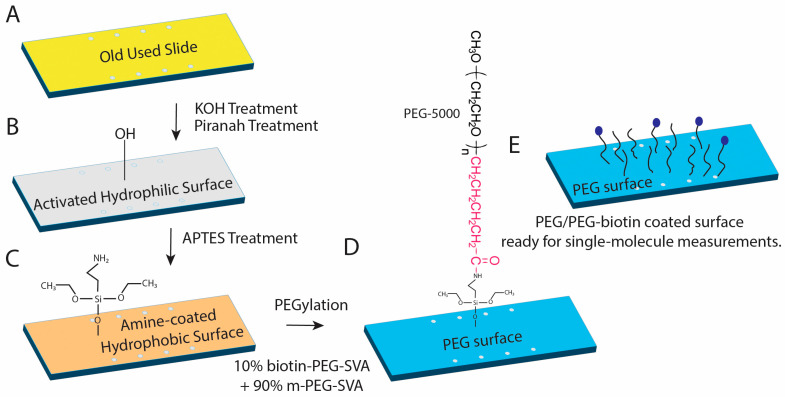
Schematic of quartz slide surface preparation and PEGylation for single-molecule fluorescence experiments. (**A**) Previously used quartz slide prior to chemical cleaning. (**B**) Activation of the glass surface by sequential KOH and piranha treatments generates a hydrophilic, hydroxyl-rich surface. (**C**) Aminosilanization with APTES introduces surface amine groups, producing an amine-coated slide suitable for subsequent functionalization. (**D**) PEGylation of the aminated surface using a mixture of methoxy-PEG–SVA (90%) and biotin-PEG–SVA (10%) yields a protein-resistant PEG layer with sparsely distributed biotin functionalities. (**E**) Final PEG/PEG-biotin–coated surface enables specific immobilization of biotinylated biomolecules via streptavidin while suppressing nonspecific adsorption, making the slide suitable for quantitative single-molecule measurements.

Quartz slides are drilled with a mini bench drill using a 1 mm diamond tip drill bit to create eight holes in a 2 × 4 pattern. Double-sided tape is used to create the channel. Double-sided tape is placed in a tabletop cutter (Silhouette Portrait 3; Silhouette America, Lindon, UT, USA) where four channels that are a rounded rectangular shape are cut onto the tape (Figure 2A). The resulting device contains four parallel, independent flow chambers, allowing multiple samples or experimental conditions to be processed and thereby increasing experimental throughput. The drilled glass slides were first thoroughly rinsed with water and methanol to ensure that no visible contaminants remained on the surface. Five slides and seven cover glasses were placed into two Coplin jars, and all subsequent cleaning steps were performed within these containers. A 1 M potassium hydroxide (KOH) solution was then added to the Coplin jars containing the slides and cover glasses, and the samples were sonicated in a bath sonicator for 30 min (Figure 3B). The KOH treatment mildly etches the glass surface, removing a molecular layer and thereby eliminating surface contaminants. Exposure to KOH was carefully limited to preserve the structural integrity of the slides and cover glasses. Following the KOH treatment, the slides and cover glasses were thoroughly rinsed with water and dried under a stream of nitrogen gas. The slides were then further cleaned by incubation in a basic piranha solution (Figure 3B). This solution was prepared by heating a mixture of 14% ammonium hydroxide (NH_4_OH) and 14% hydrogen peroxide (H_2_O_2_) until the temperature reached 70 °C. The slides and cover glasses were incubated in this solution for 30 min to remove residual organic contaminants from the glass surface. After the slides and cover, glasses were thoroughly rinsed with water and dried under a stream of nitrogen gas.

### 4.2. PEGylation

Aminosilylation was performed in 2% (*v*/*v*) APTES (1.5 mL APTES in 73.5 mL acetone) for 10 min to allow for amine functional groups to reside on the slide surface to create a positive charge (Figure 3C). This was followed by 1 min sonication in order to reduce aggregation of amine groups. This step is very important in order for single molecules to bind to the slide surface. After 1 min sonication, an additional 10 min incubation of the slides was completed to allow for complete binding of the amine groups. After this, the slides were washed thoroughly with water five times and dried with nitrogen gas. After aminosilyation, PEGylation was performed (Figure 3D). PEGylation passivates the quartz slide surface which enables specific binding of biomolecules [31,32,33]. At the beginning of slide PEGylation, freshly prepared 350 μL of 0.1 M NaHCO_3_ (84 mg/10 mL) was used to dissolve 80 mg mPEG (methoxy-polyethylene glycol) and 8 mg Biotin-PEG (biotinylated polyethylene glycol). Methoxy-PEG helps in surface passivation and reducing nonspecific binding, while Biotin-PEG introduces biotin groups that enable specific immobilization of biotin-labeled molecules through streptavidin or neutravidin binding (Figure 3E). The solution was vortexed and centrifuged at 10,000 rpm for 1 min at 4 °C. Keeping the mixture at a low temperature ensures that the reaction slows down and does not go to completion before it can be placed onto the slides. After centrifugation, 60 μL of the mixture was applied per slide and was covered with a coverslip, making sure that no bubbles were present. To incubate the slides, humid chambers were created using empty pipette tip boxes with water at the bottom that prevent slide surfaces from drying out. These slides can be incubated ≥2 h at room temperature or overnight in a humid, dark environment. Once the incubation was complete, the slide surfaces were washed extensively with Milli-Q water (12 washes in 3 × 4 cycles) to remove excess solution and dried with nitrogen gas. Following that, slide chambers were prepared by mounting the coverslips with double sticky tapes. Double-sided polyimide tape (2″ × 36 yds, 2 mil (~0.1 mm) thickness; http://www.uline.com/Product/Detail/S-17215/High-Temperature-Tape/Kapton-Tape-2-Mil-2-x-36-yds) (accessed on 24 April 2026) was used to create the channel. To prevent buffer leakage between chambers, the narrow end of a pipette tip was cut at an angle to create a flat surface, which was then used to apply uniform pressure along the tape and remove any trapped air bubbles. The same technique was used after placement of the coverslip to ensure firm, even adhesion across the device and maintain effective sealing between adjacent chambers. The assembled chambers were then vacuum-sealed in storage bags and kept at −20 °C until use.

### 4.3. Flow Chamber

The purpose of the flow chamber is to assist and facilitate the flow of samples and solutions into the chambers of the slide while an experiment is in progress. The flow chamber was fabricated from 3D printing using PLA filament (Figure 2A). The active slide was positioned between the top and bottom components of the apparatus, which were aligned and secured using six screws to ensure uniform compression and sealing. The chamber design includes eight access ports aligned with the corresponding holes in the slide. On the inlet side, each port is widened into a funnel geometry to facilitate manual fluid introduction via pipette (Figure 2B,C). Each inlet port was positioned directly above a rubber O-ring (OD = 6 mm, ID = 4 mm, thickness = 1 mm), which provides a sealed interface between the flow chamber and the slide surface. Fluid flows through the inlet port, passes through the corresponding hole in the slide, and flows across the channel to the outlet port on the opposite side (Figure 2C). The outlet was fitted with a lure that was connected to 1/16″ ID tubing. The tubing leads to a syringe mounted on a NE-1000 Syringe Pump (New Era Pump Systems Inc, Farmingdale, NY, USA; Figure 2D). Controlled withdrawal by the pump generates negative pressure, drawing solution through the channel and enabling precise fluid introduction or exchange within the chamber.

### 4.4. Oxygen-Scavenging System (OSS)

**Figure 4:**
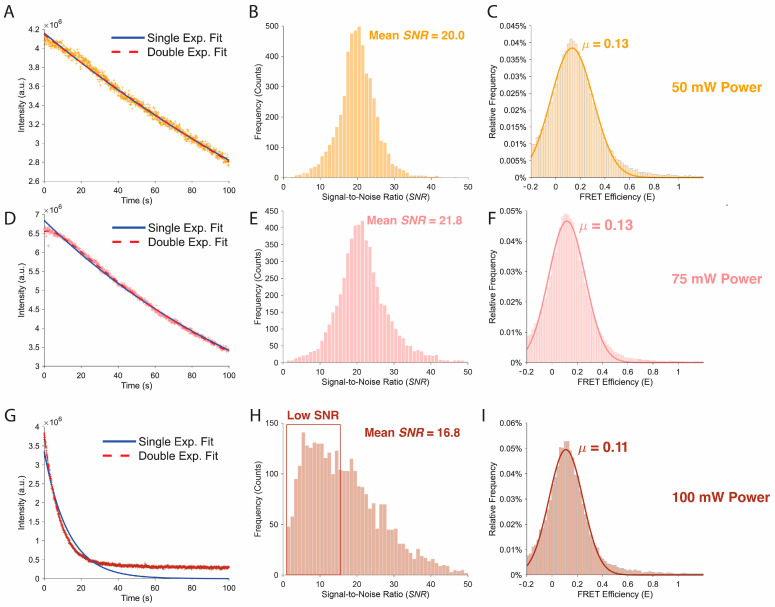
Signal quality, photobleaching behavior, and FRET distributions as a function of excitation power. (**A**,**D**,**G**) Representative fluorescence intensity trajectories under continuous illumination at 50 mW (**A**), 75 mW (**D**), and 100 mW (**G**) excitation power. Single exponential (solid blue) and double exponential (dashed red) fits are shown for comparison, highlighting changes in photobleaching dynamics with increasing laser intensity. (**B**,**E**,**H**) Corresponding distributions of signal-to-noise ratio (SNR) extracted from single molecule traces at each excitation power. Mean SNR values are indicated, with reduced SNR and increased heterogeneity observed at the highest excitation power. (**C**,**F**,**I**) FRET efficiency histograms for each excitation condition, with Gaussian fits overlaid and mean FRET efficiencies (μ) indicated. Comparable mean FRET values are observed at 50 mW and 75 mW, while higher excitation power (100 mW) results in a modest shift and broader distribution, consistent with increased noise and photophysical effects.

In single-molecule fluorescence experiments, an oxygen-scavenging system is usually added to the sample buffer to reduce dissolved molecular oxygen. The presence of oxygen can lead to photobleaching, blinking, and oxidative damage of organic fluorophores which reduces imaging time and affects signal stability. Here we used 100 mM protocatechuic acid (PCA) and 1 μM protocatechuate-3,4-dioxygenase (PCD) to remove oxygen from the solution and improve fluorophore photostability. In a PCA/PCD oxygen-scavenging system, PCA acts as the substrate and PCD is the enzyme that consumes dissolved oxygen. Together, they remove oxygen from the imaging buffer, which helps reduce oxygen-mediated photobleaching of fluorescent dyes. The PCD mixture was prepared by resuspending 8.3 mg of lyophilized PCD enzyme in 7 mL of refrigerated PCD buffer (100 mM Tris-HCl pH 8.0, 50 mM KCl, 1 mM EDTA, and 50% glycerol) and using a 0.2 μm sterile filter to remove any contaminants from the mixture. The mixture was then divided into 1.5 mL aliquots and stored at −80 °C. The PCA mixture was prepared by dissolving 0.11 g of PCA (3,4-dihydroxybenzoic acid) in 5 mL of autoclaved water. The mixture was brought to pH ~8.3 with the addition of 5 M KOH to ensure dissolution of the PCA and dilution to 7 mL with deionized water. The PCA mixture was divided into 1 mL aliquots after using a 0.2 μm sterile filter and stored at −80 °C. A PCA/PCD combination can create an extreme reducing environment sometimes which can lead to fluorophore blinking (Figure 4C) and formation of long-lived dark states. This is especially important for cyanine dyes such as Cy5, which can switch between fluorescent and non-fluorescent states during imaging. Therefore, along with PCA/PCD, a third component like Trolox is required in the OSS as a dark state quencher. The Trolox mixture was created by adding 0.2 g of Trolox into 5 mL of autoclaved water. Next, 5 M KOH was used to bring the pH of the mixture to 10–11 and to make sure that all the Trolox was fully dissolved within the mixture. Autoclaved water was added to the mixture to bring it to a total volume of 8 mL. The Trolox mixture was run through a 0.2 μm sterile filter, divided into 1 mL aliquots, and stored at −80 °C. Each mixture was aliquoted to prevent contamination. Prior to any experiment, the OSS components are thawed on ice and stored at −20 °C after use. PCD must be added last and right before the experiment is performed since it starts the reaction. We note that alternative oxygen-scavenging systems, including glucose oxidase/catalase-based formulations, have been reported to influence fluorophore blinking and photostability in single-molecule fluorescence experiment. The relative performance of these systems can depend on buffer composition, dye chemistry, and experimental conditions. A systematic comparison of oxygen-scavenging strategies was beyond the scope of this work but represents an important consideration for optimizing smFRET measurements.

## 5. Quantitative Performance Benchmarks

To evaluate the quantitative performance of the smFRET imaging platform and to maintain a good quality control between data-sets, we systematically characterized several key metrics that directly influence the accuracy and reliability of fluorescence measurements.

### 5.1. Photobleaching Statistics

Photobleaching behavior was characterized as a function of excitation laser intensity to evaluate fluorophore stability and determine excitation conditions that allow sufficiently long observation times for single-molecule measurements (Figure 4). Surface-immobilized fluorophores were imaged under three excitation powers (50 mW, 75 mW, and 100 mW) while maintaining identical acquisition parameters, including exposure time, detector settings, and imaging buffer composition. Fluorescence intensity traces were extracted from the recorded movies and averaged to evaluate the temporal decay in fluorescence resulting from photobleaching.

For each excitation condition, the ensemble fluorescence intensity was plotted as a function of time and fit using both single-exponential and double-exponential decay models to assess the underlying bleaching kinetics. At moderate excitation powers (50 mW and 75 mW), the fluorescence intensity decayed gradually over the observation period, indicating relatively slow photobleaching and stable fluorophore emission (Figure 4A,D). In these regimes, the decay curves were reasonably described by exponential models, suggesting that bleaching occurs through stochastic photochemical processes that accumulate over time.

At higher excitation powers (100 mW), a substantially faster decay in fluorescence intensity was observed (Figure 4G). The rapid initial drop in signal indicates accelerated photobleaching caused by increased excitation rates, which raise the probability of irreversible photochemical damage to the fluorophores. Under these conditions, the majority of the fluorescence signal was lost within the early portion of the acquisition window, indicating that excessive excitation power significantly reduces the usable observation time for single-molecule trajectories.

### 5.2. Signal to Noise as a Function of Laser Intensity

The signal-to-noise ratio (SNR) of the fluorescence detection system was characterized as a function of excitation laser intensity to identify the optimal operating regime for single-molecule measurements (Figure 4). Surface-immobilized fluorescent molecules were imaged over a range of excitation powers spanning the typical conditions used in smFRET experiments. For each excitation intensity, fluorescence time traces were recorded while maintaining identical acquisition parameters, including exposure time, detector gain, and imaging buffer composition, to ensure that differences in measured signal arose solely from changes in excitation power.

The fluorescence signal was defined as the mean background-corrected intensity of individual diffraction-limited spots corresponding to single molecules. To robustly estimate signal and background contributions from the intensity distribution, a percentile-based approach was employed. Specifically, the fluorescence signal was calculated from the mean intensity within the 93rd–98th percentile of the intensity distribution, representing the upper portion of the distribution dominated by single-molecule emission events. Background noise was estimated from the lower portion of the intensity distribution, defined as the 2nd–7th percentile of the recorded intensities. The extreme upper and lower 2% of the intensity range were excluded from analysis to avoid artifacts arising from transient high-intensity fluorescence bursts, detector anomalies, or dead pixels. Noise was defined as the standard deviation of the background intensity within the 2nd–7th percentile interval. The SNR was then calculated as the ratio of the mean signal intensity to the background noise level. This analysis provided a quantitative measure of detection performance across excitation powers and enabled identification of an excitation regime that maximizes the fluorescence signal while minimizing background fluctuations.

$$SNR=\frac{\mu_{intensity\;93-98th\;percentile}-\mu_{intensity\;7-2th\;percentile}}{\sigma_{intensity\;7-2th\;percentile}}$$

SNR values were determined across multiple molecules and averaged for each excitation condition. As expected, fluorescence signal increased approximately linearly with excitation intensity at low power, reflecting the proportional increase in excitation rate (Figure 4B,E). At higher laser powers, the improvement in SNR began to plateau due to increased shot noise and the onset of accelerated photobleaching (Figure 4H). These measurements allowed for the identification of an excitation regime that maximizes fluorescence signal while minimizing noise contributions and photodamage, thereby defining optimal imaging conditions for smFRET experiments. This percentile-based approach is conceptually analogous to conventional ROI-based SNR calculations, where signal and background are derived from bright and dim image regions, respectively. By excluding the extreme upper and lower ~2% of intensities, this method reduces sensitivity to outliers such as hot pixels and transient artifacts, resulting in a more robust and reproducible estimate of signal and background across imaging conditions.

### 5.3. Donor Leakage

Accurate determination of FRET efficiency requires careful correction for spectral cross-talk between the donor and acceptor detection channels. One of the primary sources of such cross-talk is donor leakage, which arises when a fraction of the donor emission spectrum overlaps with the acceptor detection window and is therefore detected in the acceptor channel. If uncorrected, this leakage contribution artificially increases the measured acceptor intensity and leads to systematic overestimation of FRET efficiency. Consequently, quantification of donor leakage is an essential calibration step for reliable smFRET measurements. To evaluate donor leakage in the present prism-TIRF system, donor-only fluorescent molecules were imaged under donor excitation while simultaneously recording signals in both the donor and acceptor detection channels. Because these molecules lack an acceptor fluorophore, any signal observed in the acceptor channel originates from spectral bleed-through of donor emission or from optical cross-talk within the detection pathway. By comparing the donor-channel intensity with the corresponding intensity detected in the acceptor channel, the fractional leakage coefficient can be determined. At all excitation powers, the FRET histograms display a well-defined peak centered near *E* ≈ 0.12 (Figure 4C,F,I), consistent with the expected low-FRET state of the control sample. Without proper calibration of the leakage coefficient, these effects can bias the calculated FRET efficiency and obscure the true structural states of the molecule. Donor leakage was measured independently and incorporated into the FRET analysis pipeline as a correction factor during trace processing. This calibration ensures that the calculated FRET efficiencies reflect true energy transfer between donor and acceptor fluorophores rather than artifacts arising from spectral overlap or detector crosstalk.

Consistent with these observations, excitation powers of 50 mW and 75 mW produced comparable signal-to-noise ratios while maintaining longer fluorescence lifetimes, whereas imaging at 100 mW resulted in a noticeable reduction in SNR and substantially faster photobleaching.

## 6. Single-Molecule Data Analysis Workflow

### 6.1. Model System and Experimental Parameters

**Figure 5:**
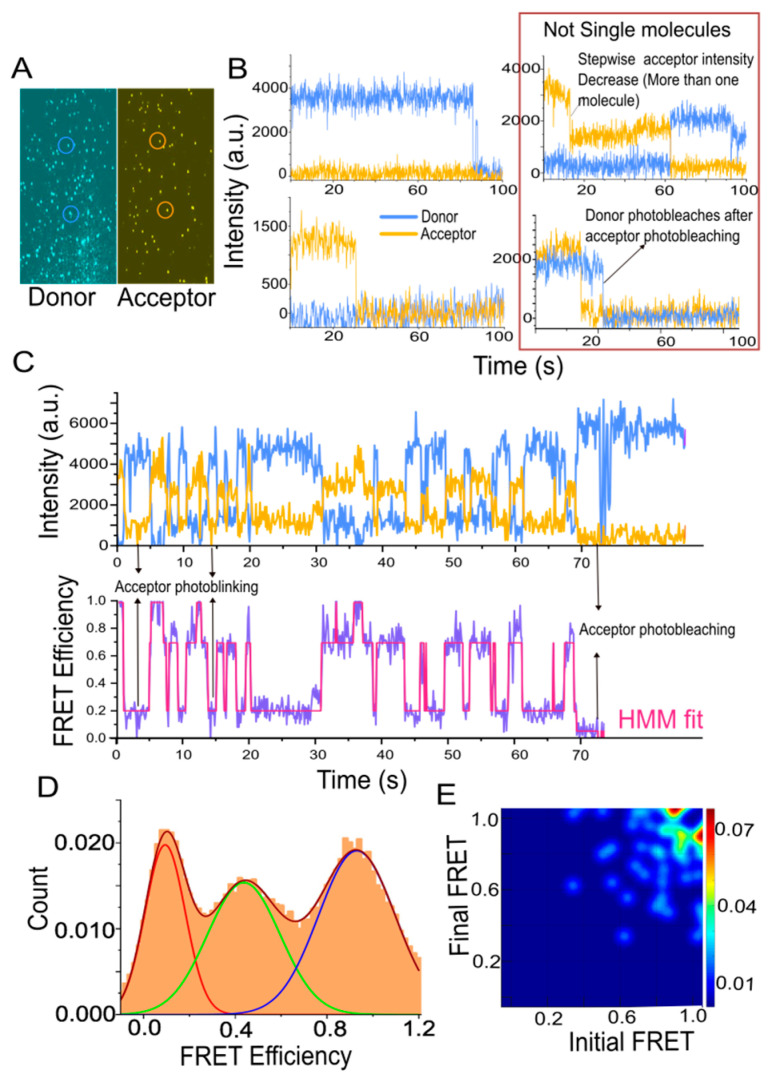
Channel registration, data processing, manual detection, and interpretation of single-molecule trajectories, hidden Markov modeling, and transition Density Analysis. (**A**) Snapshot of donor and acceptor channel showing single-molecule peaks. (**B**) Representative single-molecule traces showing only donor or acceptor. Traces showing stepwise acceptor intensity decrease or anomalous donor behavior after acceptor photobleaching should not be considered for single-molecule data analysis. Stepwise donor or acceptor intensity decrease indicates presence of more than one molecule. (**C**) Example of a single-molecule time trajectory. Data represent donor (Cy3, in blue) and acceptor (Cy5, in orange) intensity fluctuations over time (**top**), from which $E_{app}$ is calculated (FRET, purple) (**bottom**). Data representing bacterial ribosomal protein S1 mediated unfolding of RNA higher order structure, where it istransitioning between different states. The anti-correlated nature of the donor and acceptor intensity fluctuation indicates that these intensity changes are due to energy transfer. Fluorescent dyes show transition to triplet states, for example, acceptor intensity transiently drops to zero (~4 s, 15 s), before its photo bleaches completely (~70 s). (**D**) Histogram shows how each FRET states are populated. (**E**) A Transition Density Occupancy Plot (TODP), is generated using dwell time information of each FRET states.

The SM analysis platform was validated using a model nucleic acid system consisting of an RNA-G quadruplex construct labeled with a donor (Cy3). The acceptor (Cy5) fluorophore was attached to a biotinylated capture DNA strand that linked the construct to the surface as well as reported on conformational rearrangements. RNA/DNA duplex constructs were annealed and immobilized on PEGylated quartz slides via biotin–streptavidin linkage at low surface density (<0.1 molecules/µm^2^) to ensure diffraction-limited spatial separation. Experiments were carried out in a Tris-based imaging buffer (50 mM Tris-HCl, 50 mM NaCl) supplemented with an oxygen-scavenging system (PCA/PCD, Trolox) to suppress photobleaching and blinking. Data were acquired under continuous excitation (50–75 mW) with a 100 ms frame integration. Under these conditions, individual molecules exhibited anticorrelated donor–acceptor intensity fluctuations and stochastic transitions between discrete FRET states. Representative time traces, FRET trajectories, and population distributions are shown in Figure 5.

### 6.2. Data Acquisition and Channel Registration

An accurate spatial registration between the donor and acceptor channels was obtained through an overlap map, which was generated using surface-immobilized 200 nm fluorescent microspheres (Invitrogen, TetraSpeck™ Microspheres, T7280, Thermo Fisher Scientific, Waltham, MA, USA) that emit strongly in all detection channels. When required, the emission optics were manually adjusted to optimize channel alignment. Matched reference points were then used by an automated algorithm to calculate a coordinate transformation between the two channels, modeled as a 16-parameter bivariate quadratic function. This transformation accounts for translational offset, rotation, scaling mismatches, and optical distortion. The resulting overlap map was subsequently applied to single-molecule movies to register donor and acceptor coordinates to extract single-molecule time trajectories (Figure 5A).

### 6.3. Automated Initial Data Processing

To convert movie files into single-molecule time trajectories, each movie, a maximum-intensity projection was generated from selected range frames to enhance the signal-to-noise ratio for peak detection. Donor and acceptor channel images were then separated and processed independently. Peaks were identified using local-noise thresholding, and curvature filtering to detect spatially localized fluorescence spots while rejecting nonspecific or poorly defined features. Peaks located near image boundaries were excluded to eliminate poor illumination-related errors. The overlap map, generated using fluorescent beads, was then applied to transform acceptor-channel coordinates into the donor-channel reference frame, enabling peaks to be classified as common to both channels or unique to either donor or acceptor channels. Depending on the analysis objective, users could select common peaks only, donor-only peaks, acceptor-only peaks, or all detected peaks for subsequent trace generation. Donor and acceptor fluorescence intensities were then extracted frame by frame at the selected positions at their respective channels. Local background levels were estimated by masking the signal region surrounding each molecule and sampling the adjacent area. Furthermore, automated downstream analysis prescreened trajectories using objective quality-control criteria to exclude molecules that were too dim, too bright, excessively noisy, or that photobleached prematurely. Finally, the accepted donor and acceptor trajectories and their corresponding coordinates were written to data files.

### 6.4. Manual Detection and Interpretation of Single-Molecule Trajectories

To minimize user bias, initial trajectory selection was supported by automated prescreening using objective criteria such as intensity thresholds, noise levels, and photobleaching behavior. Manual inspection was used as a secondary validation step to confirm single-molecule characteristics. The standardized data structure produced by this workflow is compatible with emerging machine learning approaches for automated trajectory classification and state identification, which can be integrated in future implementations to further improve reproducibility and scalability. Despite automated prescreening, manual inspection of single-molecule time trajectories is essential to establish that each trace originates from a bona fide single molecule (Figure 5B). True single-molecule trajectories are identified by characteristic photophysical signatures, including anticorrelated donor and acceptor intensity fluctuations accompanied by a stable total fluorescence signal prior to photobleaching, and a single-step, irreversible photobleaching event. The presence of multiple stepwise intensity decreases (Figure 5B), non-conserved total intensity or anomalous donor behavior following acceptor photobleaching (Figure 5B) is indicative of contributions from multiple emitters or other non-single-molecule effects and necessitates exclusion from further analysis.

Manual screening also enables the identification of artifacts that are difficult to robustly detect algorithmically. Common artifacts include reversible transient colocalization of multiple molecules within the diffraction-limited spot, surface-induced fluorescence fluctuations, and atypical photo physics arising from dye impurities or defective constructs. Loss of acceptor fluorescence in a reversible way, often called blinking (Figure 5C), is a common artifact and can easily be mistaken for a real structural transition, such as large conformational change or macromolecular unfolding. In general, if the FRET signal drops suddenly to the donor-only level, with apparent $E_{FRET}$ = 0, it should first be suspected as dye blinking rather than a true low-FRET state. However, blinking can be ruled out if the low FRET states persist even in the presence of triplet state quenchers like Trolox and if direct acceptor excitation confirms that the acceptor dye is still active. Such criteria provide a framework for distinguishing true molecular dynamics from experimental or photophysical artifacts. Manual selection further allows trajectory specific background subtraction. FRET efficiency is calculated as EFRET=IA(IA+ID), where $I_{D}$ and $I_{D}$ represent donor and acceptor intensities, respectively. Different time segments from traces can be manually selected; these selected regions are exported containing information of time, donor intensity, acceptor intensity, and FRET values, and they are accumulated into a normalized cumulative FRET histogram (Figure 5D).

### 6.5. Hidden Markov Modeling and Transition Density Analysis

Individual single-molecule time trajectories in equilibrium contain important kinetics information of the system through dwell times in different conformational states [34]. For example, if stochastic transitions are happening in a two-state system, the transition rates can be determined by fitting exponential decay curves to the dwell time distribution of each state or if the transitions are fast to determine precise dwell times, the rates can instead be estimated using autocorrelation analysis together with the equilibrium populations of the states [35]. There can be complex transitions involving several distinct conformational states. In such cases, Hidden Markov Modeling (HMM) can be used for an unbiased estimation of number of populated FRET (conformational) states [36], rates of interconversion between them, and dwell time for each state (Figure 5C). Software like vbFRET (v2.5 10 July 2009) [37], HaMMy (version 1.0.0, 11 June 2021) [36], and QuB (ver. 2.0.0.22) [38] can be used for Hidden Markov Model analysis by representing single-molecule dynamics as a state model and using maximum-likelihood algorithms to recover the most probable state sequence, transition rates, and state occupancies from noisy time traces. Transition Occupancy Density Plots (TDPs) can be used to show initial and final FRET values for each transition identified by the Hidden Markov Model [36,39,40], which represents a concise method to summarize the transition data (Figure 5E). For the model nucleic acid system studied here, analysis of individual trajectories using hidden Markov modeling revealed transitions between multiple discrete FRET states, with dwell-time distributions consistent with a multi-state kinetic model (Figure 5C–E). These results demonstrate the capability of the platform to resolve conformational dynamics and extract quantitative kinetic information from single-molecule trajectories.

## 7. Conclusions

Our work presents a standardized prism-based total internal reflection fluorescence (TIRF) platform for quantitative single-molecule fluorescence studies of biomolecular dynamics. By integrating a home-built prism-based excitation geometry with a commercial inverted microscope platform, this implementation provides a practical balance between optical flexibility, experimental robustness, and accessibility. Prism-based TIRF is primarily advantageous for surface-tethered biomolecular assays [16,23,26,27] because it allows selective excitation within a thin region near the slide–solution interface [23,24], reduces background fluorescence [22,25], and supports imaging under active flow conditions, which are often required for kinetic [28] and ligand-dependent experiments. The robust nature of the design also allows adaptation to different fluorophore combinations, laser lines, emission filters, and biomolecular systems [4,23,25,27,41], which makes the platform broadly useful for studies involving nucleic acids, proteins, protein–nucleic acid complexes, and multi-component assemblies [12,42,43,44,45,46]. An important aspect of the platform is reproducibility and robustness. Previous studies have shown that these microscopes are important tools for visualizing macromolecular dynamics and can be adapted for a broad range of biological applications [5,16,23,25,26,27,31,41]. However, single-molecule fluorescence experiments are quite sensitive to optical alignment [4,18,19,24], channel registration [21,30], surface quality [33], fluorophore choice [4], background fluorescence [22], and data-acquisition conditions [21,28,29,30,32]. Our standardized guides reduce dependence on trial-and-error-based optimization efforts and establish practical criteria for robust and reproducible instrument performance. Measurements of signal-to-noise ratio, background fluorescence, photobleaching behavior, field uniformity, and donor–acceptor crosstalk provide quantitative benchmarks that make the system suitable for reliable single-molecule studies.

Our platform further demonstrates the use of pTIRF microscopy to study biological systems including dynamic nucleic acids like Holiday Junctions. Since single-molecule fluorescence measurements directly capture molecule-to-molecule heterogeneity and time-dependent transitions, this approach provides mechanistic information that is often obscured in ensemble-averaged experiments. Although demonstrations here use nucleic acid systems due to their well-established behavior in smFRET, the same framework is directly applicable to more complex biological problems, including protein–protein interactions and protein–nucleic acid assemblies. Such systems can be readily incorporated using established labeling and immobilization approaches, enabling investigation of conformational dynamics and interaction mechanisms in biologically relevant contexts. Extension to these applications represents a straightforward and important direction for future studies.

Overall, our standardized pTIRF platform provides an important foundation for high-quality quantitative single-molecule fluorescence measurements. Its modular design can be adapted to different fluorophore pairs, biomolecular targets, and experimental requirements, while the calibration and benchmarking workflow supports reproducibility across users and experiments.

## Figures and Tables

**Table 1:** Optical component list and notes.

No.	Item	Notes	Catalog ID	Vendor	Quantity
1	Optical table	3′ × 5′ × 4″ optical tabletop w/TMC pneumatic isolators/tie bar	NA	Newport (Irvine, CA, USA)	1
2	Microscope body	Inverted Ix83 microscope with Z-drift correction	IX83	Olympus (Hachioji, Tokyo, Japan)	1
3	Sample stage	Motorized XY stage	ProScan H100BX	Prior	1
4	60X objective	Oil objective, OFN26.5, NA1.42, WD0.15 mm (about 0.01 in)	UPLXAPO60XO	Olympus (Hachioji, Tokyo, Japan)	1
5	Camera	6.5 µm × 6.5 µm pixel size, quantum efficiency ~80%	OH-C14440-20UP-KIT	Hamamatsu (Hamamatsu City, Japan)	1
6	ND filters	0.4 optical density, 1.0 in. diameter	5234NF	New Focus (Andover, MA, USA)	5
7	532 laser	Wavelength 532 ± 2 nm, output power 10 mW–0.5 W; beam diameter 1.5 ± 0.05 mm (to excite Cy3)	Sapphire 532 FPT FT	Coherent (Saxonburg, PA, USA)	1
8	FC/APC fiber connectors	Single mode, 81 μm Bore, ceramic ferrule, 900 μm Boot	30080K2	Thor Labs Inc. (Newton, NJ, USA)	4
9	637 laser	Wavelength 637 nm, output power 140 mW; beam diameter 0.70 ± 0.1 mm (to excite Cy5)	OBIS LX 637	Coherent (Saxonburg, PA, USA)	1
10	Laser shutter and driver	1-inch aperture	VCM-D1	VINCENT ASSOCIATES	1
11	Pedestal pillars and mechanical posts	Ø1″, 1/4″-20 taps, L = different lengths	-	Thor Labs Inc. (Newton, NJ, USA)	30
12	Kinematic mirror mount	Ø1″ optics	KM100	Thor Labs Inc. (Newton, NJ, USA)	10
13	Broadband dielectric mirror	Ø1″ broadband dielectric mirror, 400–750 nm, 10 pack	BB1-E02-10	Thor Labs Inc. (Newton, NJ, USA)	1
14	Positive achromatic doublets	f = 180 mm, Ø2″ achromatic doublet, ARC: 400–700 nm	AC508-180-A	Thor Labs Inc. (Newton, NJ, USA)	1
15	Positive achromatic doublets	f = 100 mm, Ø1″ achromatic doublet, ARC: 400–700 nm	AC254-100-A	Thor Labs Inc. (Newton, NJ, USA)	1
16	XYZ Linear Stage, ULTRAlign	25 mm travel, crossed-roller, M4, M6	M-462-XYZ-M	Newport (Irvine, CA, USA)	1
17	Periscope assembly	Mirrors not included	RS99	Thor Labs Inc. (Newton, NJ, USA)	1
18	Dovetail linear stage for prism holder	1.0 in. travel, fast-drive 20 TPI screw, 1/4–20 threads	TSX-1D	Newport (Irvine, CA, USA)	1
19	Prism	UV FS Pellin–Broca prism 11 × 20 × 6.4 mm	325-1206	EKSMA Optics (Vilnius, Lithuania)	1
20	Dichroic beamsplitter	26 × 38 × 2 mm	T556lpxr	Chroma Technology Corp (Bellows Falls, VT, USA)	1
21	Long pass Dichroic Beamsplitter	T635lpxr-UF2, 2 mm thickness; ≤0.5 waves/inch peak-to-valley flatness	T635lpxr	Chroma Technology Corp (Bellows Falls, VT, USA)	2
22	Notch filter	Deeply attenuates 635 nm while transmitting other wavelengths	ZET635NF	Chroma Technology Corp (Bellows Falls, VT, USA)	1
23	Long pass filter	Transmits light above 537 nm and blocks shorter wavelengths	RET537lp	Chroma Technology Corp (Bellows Falls, VT, USA)	1
24	Adjustable mechanical slit	One-way micrometer adjustable optical slit, single adjustable slit 20 mm	Lsxf2-20	Canglan Technology Store (Digital Storefront)	1
25	Iris diaphragm	Lever-actuated, SM-threaded, SM1 (1.035″-40), aperture (Min): Ø1.0 mm, aperture (Max): Ø12.0 mm	SM1D12	Thor Labs Inc. (Newton, NJ, USA)	2
26	Kinematic mounts	1″ square optics	KM100S	Thor Labs Inc. (Newton, NJ, USA)	2

## Data Availability

All analysis codes used in this study are available from the corresponding author upon reasonable request.
